# Supplementary material for: Scabies epidemiology in health care centers for refugees and asylum seekers in Greece
Source: PLoS Negl Trop Dis. 2022 Jun 22;16(6):e0010153. doi: 10.1371/journal.pntd.0010153 (PMC9255768; doi:10.1371/journal.pntd.0010153)
Supplement: S1 Table — (DOCX) [file pntd.0010153.s001.docx]

| Monthly time periods | Total consultations | Scabies cases | Percentage of scabies cases to total visits (%) | Respiratory infections cases | Gastrenteritis no blood cases | Rash with fever cases | Tuberculosis cases |
| --- | --- | --- | --- | --- | --- | --- | --- |
| Jun-16 | 18400 | 102 | 0.55 | 774 | 610 | 108 | 8 |
| Jul-16 | 36550 | 170 | 0.47 | 1773 | 1139 | 186 | 6 |
| Aug-16 | 35967 | 158 | 0.44 | 1263 | 695 | 68 | 13 |
| Sep-16 | 41167 | 237 | 0.58 | 1255 | 562 | 170 | 29 |
| Oct-16 | 30615 | 356 | 1.16 | 975 | 301 | 204 | 15 |
| Nov-16 | 28770 | 201 | 0.70 | 893 | 208 | 149 | 16 |
| Dec-16 | 27524 | 190 | 0.69 | 775 | 193 | 120 | 7 |
| Jan-17 | 20025 | 164 | 0.82 | 886 | 111 | 45 | 4 |
| Feb-17 | 17476 | 127 | 0.73 | 756 | 90 | 41 | 9 |
| Mar-17 | 22644 | 166 | 0.73 | 716 | 177 | 54 | 14 |
| Apr-17 | 17274 | 83 | 0.48 | 404 | 164 | 21 | 3 |
| May-17 | 14917 | 78 | 0.52 | 269 | 90 | 9 | 4 |
| Jun-17 | 19329 | 115 | 0.59 | 453 | 118 | 5 | 6 |
| Jul-17 | 11917 | 63 | 0.53 | 351 | 128 | 10 | 6 |
| Aug-17 | 12517 | 43 | 0.34 | 302 | 90 | 13 | 14 |
| Sep-17 | 19143 | 82 | 0.43 | 544 | 150 | 29 | 14 |
| Oct-17 | 15250 | 97 | 0.64 | 438 | 80 | 18 | 9 |
| Nov-17 | 17166 | 129 | 0.75 | 437 | 110 | 26 | 29 |
| Dec-17 | 17783 | 69 | 0.39 | 423 | 85 | 19 | 12 |
| Jan-18 | 13178 | 94 | 0.71 | 524 | 88 | 15 | 10 |
| Feb-18 | 15514 | 93 | 0.60 | 697 | 110 | 28 | 6 |
| Mar-18 | 21311 | 130 | 0.61 | 609 | 123 | 51 | 24 |
| Apr-18 | 18083 | 109 | 0.60 | 512 | 153 | 48 | 46 |
| May-18 | 18293 | 152 | 0.83 | 486 | 264 | 47 | 58 |
| Jun-18 | 20660 | 118 | 0.57 | 451 | 350 | 64 | 29 |
| Jul-18 | 17175 | 130 | 0.76 | 466 | 246 | 43 | 54 |
| Aug-18 | 20356 | 187 | 0.92 | 513 | 216 | 24 | 30 |
| Sep-18 | 18179 | 209 | 1.15 | 543 | 209 | 20 | 12 |
| Oct-18 | 17372 | 171 | 0.98 | 483 | 243 | 17 | 14 |
| Nov-18 | 19846 | 181 | 0.91 | 568 | 232 | 41 | 14 |
| Dec-18 | 15183 | 130 | 0.86 | 274 | 181 | 45 | 19 |
| Jan-19 | 18464 | 117 | 0.63 | 614 | 262 | 58 | 15 |
| Feb-19 | 21865 | 306 | 1.40 | 463 | 215 | 105 | 25 |
| Mar-19 | 21891 | 270 | 1.23 | 457 | 170 | 227 | 27 |
| Apr-19 | 16785 | 267 | 1.59 | 418 | 143 | 225 | 14 |
| May-19 | 19653 | 326 | 1.66 | 571 | 155 | 115 | 14 |
| Jun-19 | 18536 | 235 | 1.27 | 372 | 202 | 44 | 22 |
| Jul-19 | 20649 | 248 | 1.20 | 466 | 336 | 22 | 33 |
| Aug-19 | 22452 | 246 | 1.10 | 385 | 354 | 19 | 73 |
| Sep-19 | 19467 | 208 | 1.07 | 349 | 365 | 14 | 24 |
| Oct-19 | 25222 | 517 | 2.05 | 752 | 542 | 31 | 39 |
| Nov-19 | 24717 | 400 | 1.62 | 843 | 528 | 62 | 15 |
| Dec-19 | 26808 | 744 | 2.78 | 1356 | 632 | 83 | 36 |
| Jan-20 | 29259 | 1663 | 5.68 | 1464 | 362 | 39 | 39 |
| Feb-20 | 26800 | 958 | 3.57 | 1091 | 255 | 44 | 28 |
| Mar-20 | 17083 | 292 | 1.71 | 532 | 126 | 20 | 8 |
| Apr-20 | 14366 | 335 | 2.33 | 250 | 95 | 30 | 5 |
| May-20 | 25299 | 661 | 2.61 | 289 | 218 | 43 | 4 |
| Jun-20 | 22357 | 586 | 2.62 | 194 | 380 | 35 | 8 |
| Jul-20 | 23549 | 405 | 1.72 | 274 | 332 | 19 | 3 |
